# The Sacred Connection of the Feet (Te Tuhono Tapu o ngā Waewae): The Cultural and Spiritual Impact of Diabetes‐Related Lower Limb Amputation Among Māori

**DOI:** 10.1002/jfa2.70167

**Published:** 2026-05-20

**Authors:** Cynthia Otene, Belinda Ihaka, Matthew R. Carroll

**Affiliations:** ^1^ Department of Podiatry School of Allied Health Auckland University of Technology Auckland New Zealand

**Keywords:** amputation, diabetic foot, foot screening, health equity, Māori health, podiatry services

## Abstract

Diabetes prevalence in Aotearoa New Zealand is rising rapidly, disproportionately affecting Māori communities and contributing to significantly higher rates of diabetes‐related lower limb amputations. These inequities reflect systemic issues such as colonisation, racism, and limited access to culturally safe care rather than clinical factors alone. Embedding tikanga (Māori customs) within podiatry foot screening offers a pathway to culturally responsive practice that honours Māori spiritual, physical, and cultural well‐being. Integrating Rongoā Māori (traditional healing) and whānau‐centred approaches alongside biomedical care can improve engagement and outcomes, whereas marae‐based and community‐led models enhance accessibility and trust. Workforce development in cultural safety is essential to uphold Te Tiriti o Waitangi obligations of equity, partnership, and protection. Culturally grounded podiatry services not only reduce the risk of amputation but also restore connections to tūrangawaewae (place of belonging) and whakapapa (genealogy), ensuring that care is meaningful and effective for Māori communities.

## 1 Introduction

Lower limb amputation has cultural and spiritual consequences for Māori, making it more than just a medical occurrence. Waewae (feet) serve as sacred links to the whenua (land), anchoring one's tūrangawaewae (the place of belonging), through whakapapa (genealogy). Diabetes‐related lower limb amputations can sever these connections, making culturally responsive preventative care essential to uphold Māori identity, well‐being, and to meet legislation obligations under Te Tiriti o Waitangi [1]. For this commentary, we define podiatry foot screening to encompass the early detection of complications, comprehensive foot care, patient education, and multidisciplinary management of a person with diabetes. National frameworks standardise equity assessment in podiatry foot screening, and although these frameworks are available, the prevalence rates of diabetes‐related lower limb amputations amongst Māori continue to rise [2]. Therefore, providing podiatry foot screening responsive to the cultural needs of Māori will have a positive effect on diabetes‐related lower‐limb amputation rates.

## 2 Māori Cultural Identity and Diabetes

Waewae are deeply sacred to Māori, serving as physical and spiritual anchors to Papatūānuku (Earth Mother). Therefore, waewae are the physical connection to whenua and the spiritual connection to Papatūānuku. The concept of whenua further embodies the enduring spiritual bond between people and the land. The placenta is buried in ancestral land at birth, symbolising one's ties to the land from birth to death. Consequently, the removal of any part of a limb through amputation would be viewed by Māori as more than a medical event but a disruption to this sacred connection. For many Māori, returning an amputated limb for ritual burial on ancestral land honours whakapapa, mauri (life force), and tapu (sacredness), safeguarding spiritual and cultural integrity [3]. Integrating this understanding into podiatry foot screening aligns care holistically, acknowledging the spiritual significance of waewae.

Diabetes prevalence in Aotearoa New Zealand is increasing rapidly, with more than 270,000 people currently affected and projections indicating a 90% rise by 2044 [4]. Māori are over three times more likely than non‐Māori to develop diabetes, a disparity that is evident in markedly higher rates of diabetes‐related lower limb amputations [5]. These elevated amputation rates are not solely due to clinical factors but reflect deeper systemic issues, including colonisation, racism, and inequitable access to preventative care [6]. Collectively, these factors contribute to the disproportionate burden of diabetes‐related amputations within Māori communities. In 2019, Māori accounted for 20% of all diabetes‐related lower limb amputations in Aotearoa New Zealand, despite comprising only 17% of the population [7]. Gurney et al. [5] reported that Māori faced a 65% greater risk of above‐knee amputation compared with non‐Māori, whereas Garrett and Grey [2] found that 65% of Māori admitted for diabetes‐related amputations had not seen a podiatrist in the 3 months preceding their first amputation.

Multiple factors contribute to the risk of diabetes‐related lower limb amputations, including ethnicity, social determinants, glycaemic control, peripheral neuropathy, peripheral vascular disease, foot deformities, and delayed wound healing [8]. Access to culturally safe diabetes foot care can significantly improve outcomes; however, such access remains challenging for Māori in rural and urban areas [2, 9]. This underscores the importance of timely podiatry foot screening and multidisciplinary care teams, which are essential for reducing amputation risk [10]. Crucially, podiatric screening and management must not only be accessible to Māori communities but also culturally aligned to uphold and support Māori identity.

## 3 Redressing the Destruction of Whakapapa

In 2019, the Waitangi Tribunal inquiry into the government's response to Māori health inequities confirmed longstanding failures to uphold Te Tiriti o Waitangi obligations [1]. The Health Services and Outcomes Inquiry (WAI 2575) highlighted that these breaches have contributed to entrenched inequities and poor health outcomes for Māori. These findings emphasise the need for podiatrists to dismantle systemic barriers and prioritise Māori‐led solutions that restore equity within podiatry screening and management services. However, understanding and application of tikanga Māori, cultural practices, and values‐based principles vary across the podiatry workforce. This variation is better understood as a systems‐level issue, arising from inconsistent integration of these principles within podiatry education, professional standards, and workforce development processes, which have historically been structured within Western knowledge frameworks [11]. The focus must therefore shift to actively exploring how Māori cultural understandings can inform and support podiatrists in embedding Māori world views into practice.

Tikanga is intrinsic to Māori identity and practice, with dynamic values and customs that include manaakitanga (hospitality), whanaungatanga (kinship), utu (reciprocation), ea (restoration), mana (prestige), tapu (sacred), and noa (neutrality), each helping to guide the lives of Māori [12]. Tikanga can be seen as lore (a body of traditions) to Māori, and any disruption could affect the natural balance of tapu and noa that impacts a person's mana and whanaungatanga. Tikanga practices, therefore, allow reconciliation through ea and utu rather than pure punitive actions to achieve harmony of the living (physical/cultural connection) and non‐living (spiritual connection) worlds [12]. In the Māori language, ‘tika’ translates to ‘right’ or ‘correct’ and ‘nga’ is the plural for the word ‘the’. In this form, tikanga can be seen as ‘way(s) of behaving correctly’. If, however, a definite meaning had to be attributed to tikanga, one could say that it is how Māori ancestors lived their lives, so for Māori today, these ways of living, or tikanga, are seen as everyday ways of being.

In podiatry practice, tikanga Māori can inform clinical interactions, decision‐making, and service delivery in practical and contextual ways. Principles such as whanaungatanga may be reflected in relationship‐building, attentive listening, the use of appropriate greetings or place names in te reo Māori, and the meaningful involvement of whānau in assessment and treatment discussions, when desired. Manaakitanga can shape respectful communication, flexibility in appointment structures, and attention to patient dignity and comfort across routine care, wound management, and long‐term treatment planning. Recognition of wairua as a dimension of well‐being may involve creating clinical environments in which spiritual values are acknowledged and respected, such as allowing space for karakia where patients or whānau wish to undertake this and ensuring clinical processes do not undermine spiritual or cultural safety [13, 14]. Awareness of tapu and noa may further guide sensitive handling of the body and bodily materials, prompting careful discussion and support for tikanga consistent preferences where requested. The use of te reo Māori within clinical encounters, such as correct pronunciation of Māori names, use of commonly understood kupu (words), or providing space for patients to express themselves in te reo Māori if they choose, can contribute to engagement and cultural safety without requiring practitioner fluency [15]. At a service level, tikanga‐informed approaches also include delivering podiatry services in community‐based or Indigenous‐led settings, such as marae or rural clinics, which have been shown to enhance access, foster trust, and support sustained engagement for Māori [16, 17].

## 4 Cultural Safety and Workforce Development

Workforce development in podiatry must move beyond narrow notions of cultural competence towards a framework of cultural safety, which more effectively addresses Māori health inequities in Aotearoa New Zealand. As Curtis et al. [18] argue, competence‐based approaches risk becoming static and skills‐focused, obscuring power relations, institutional responsibility, and the lived experiences of Māori. In contrast, cultural safety emphasises critical self‐reflection, examination of power, and accountability at both practitioner and organisational levels, with safety defined by those receiving care. Cultural safety initiatives grounded in Māori realities also develop transferable capabilities, including critical consciousness, reflexivity, and relational practice, which enhance care for other populations underserved by mainstream health systems. Investment in culturally safe workforce development, therefore, supports both Māori health equity and broader system improvement, aligning with Te Tiriti o Waitangi obligations and the Pae Ora (Healthy Futures) Act 2022 [19].

## 5 Conclusion

The disconnection caused by diabetes‐related lower limb amputation can represent a profound separation from one's tūrangawaewae (place of belonging) and ancestral identity. To better serve Māori, podiatry foot screening should be guided by tikanga, ensuring culturally responsive practice that acknowledges the spiritual, physical, and cultural dimensions of well‐being. Supporting these values requires comprehensive cultural safety and workforce development within podiatry, which is essential for upholding the obligations of Te Tiriti o Waitangi. Embedding tikanga within podiatric foot screening and management affirms Māori spiritual and cultural integrity while bridging gaps in understanding for non‐Māori practitioners.

## Author Contributions

**Cynthia Otene:** conceptualization, writing – original draft, writing – review and editing. **Belinda Ihaka:** conceptualization, writing – review and editing, supervision. **Matthew R. Carroll:** conceptualisation, writing – review and editing, project administration, supervision.

## Funding

This manuscript is a component of a larger study supported by funding from the New Zealand Health Research Council Māori Health Research PhD Scholarship and the Ngā Pae o te Māramatanga PhD Doctoral Scholarship Grant. The funders had no role in the conception, development, or writing of this manuscript.

## Conflicts of Interest

The authors declare no conflicts of interest.

## Data Availability

Data sharing not applicable to this article as no datasets were generated or analysed during the current study.

## References

[1] Waitangi Tribunal , Hauora: Report on Stage One of the Health Services and Outcomes Kaupapa Inquiry (WAI 2575) (Health Services and Outcomes, 2019), https://www.waitangitribunal.govt.nz/en/inquiries/kaupapa‐inquiries/health‐services‐and‐outcomes.

[2] M. Garrett and S. Gray , “Audit of Diabetes‐Related Lower Extremity Amputations in the Northern Region of New Zealand 2013–2016,” New Zealand Medical Journal 137, no. 1598 (2024): 44–54, 10.26635/6965.6045. 38963930

[3] M. Hēnare . “Tapu, Manu, Mauri, Hau, Wairua: A Māori Philosophy of Vitalism and Cosmos,” in Indigenous Spiritualities at Work: Transforming the Spirit of Enterprise [Internet], ed. C. Spiller and R. Wolfgramm (Emerald Publishing Limited, 2015), 77–98, 10.1108/978-1-68123-157-0.

[4] A. Teng , J. Stanley , J. Krebs , et al., “Projected Increases in the Prevalence of Diabetes Mellitus in Aotearoa New Zealand, 2020–2044,” New Zealand Medical Journal 138, no. 1608 (2025): 94–106, 10.26635/6965.6500. 39847739

[5] J. K. Gurney , J. Stanley , S. York , and D. Sarfati , “Regional Variation in the Risk of Lower‐Limb Amputation Among Patients With Diabetes in New Zealand,” ANZ Journal of Surgery 89, no. 7–8 (2019): 868–873, 10.1111/ans.15079. 30920078

[6] J. Reid , K. Taylor‐Moore , and G. Varona , “Towards a Social‐Structural Model for Understanding Current Disparities in Maori Health and Well‐Being,” Journal of Loss & Trauma 19, no. 6 (2014): 514–536, 10.1080/15325024.2013.809295.

[7] S. Crengle , G. Davie , J. Whitehead , B. De Graaf , R. Lawrenson , and G. Nixon , “Mortality Outcomes and Inequities Experienced by Rural Māori in Aotearoa New Zealand,” Lancet Regional Health. Western Pacific 28 (2022): 100570, 10.1016/j.lanwpc.2022.100570. 36042896 PMC9420525

[8] V. Blanchette , J. Patry , M. Brousseau‐Foley , et al., “Diabetic Foot Complications Among Indigenous Peoples in Canada: A Scoping Review Through the PROGRESS‐PLUS Equity Lens,” Frontiers in Endocrinology 14 (2023): 1177020, 10.3389/fendo.2023.1177020. 37645408 PMC10461566

[9] E. Beeler , A. Brenton‐Rule , and M. Carroll , “Recruitment and Retention of the Rural Podiatry Workforce in Aotearoa New Zealand: A Qualitative Descriptive Study of Podiatrist Perceptions,” Journal of Foot and Ankle Research 15, no. 1 (2022): 58, 10.1186/s13047-022-00562-3. 35945617 PMC9361604

[10] P. Z. Zimmet , “Diabetes and Its Drivers: The Largest Epidemic in Human History?,” Clinical Diabetes and Endocrinology 3, no. 1 (2017): 1, 10.1186/s40842-016-0039-3. 28702255 PMC5471716

[11] H. Carel and I. J. Kidd , “Epistemic Injustice in Healthcare: A Philosophical Analysis,” Medicine, Health Care and Philosophy 17, no. 4 (2014): 529–540, 10.1007/s11019-014-9560-2. 24740808

[12] H. M. Mead , Tikanga Maori (Revised Edition): Living by Maori Values (Huia Publishers, 2016).

[13] B. J. Wilson , F. A. S. Bright , C. Cummins , H. Elder , and N. M. Kayes , “‘The Wairua First Brings You Together’: Māori Experiences of Meaningful Connection in Neurorehabilitation,” Brain Impairment 23, no. 1 (2022): 9–23, 10.1017/BrImp.2021.29.

[14] B. Marques , C. Freeman , L. Carter , and M. Pedersen Zari , “Conceptualising Therapeutic Environments Through Culture, Indigenous Knowledge and Landscape for Health and Well‐Being,” Sustainability 13, no. 16 (2021): 9125, 10.3390/su13169125.

[15] T. H. R. Karaka‐Clarke , A. Macfarlane , and J. Fletcher , “Approaches to Teaching and Learning a Second Language Online,” Waikato Journal of Education 28, no. 1 (2023): 109–123, 10.15663/wje.v28i1.1000.

[16] B. Ihaka , K. Rome , and H. Came , “Diabetes Podiatry Services for Māori in Aotearoa: A Step in the Right Direction?,” Journal of Foot and Ankle Research 15, no. 1 (2022): 59, 10.1186/s13047-022-00564-1. 35945591 PMC9361539

[17] E. Curtis , R. Jones , E. Willing , et al., “Indigenous Adaptation of a Model for Understanding the Determinants of Ethnic Health Inequities,” Discover Social Science and Health 3, no. 1 (2023): 10, 10.1007/s44155-023-00040-6.

[18] E. Curtis , R. Jones , D. Tipene‐Leach , et al., “Why Cultural Safety Rather Than Cultural Competency Is Required to Achieve Health Equity: A Literature Review and Recommended Definition,” International Journal for Equity in Health 18, no. 1 (2019): 174, 10.1186/s12939-019-1082-3. 31727076 PMC6857221

[19] New Zealand Government , Pae Ora (Healthy Futures) Act 2022 (2022), https://www.legislation.govt.nz.

